# Stimuli-sensitive hydrogels: A novel ophthalmic drug delivery system

**DOI:** 10.4103/0301-4738.71677

**Published:** 2010

**Authors:** Vinod Singh, S S Bushetti, Raju Appala, Adil Shareef, Syed S Imam, Mamta Singh

**Affiliations:** Department of Pharmaceutics & Pharmacology, SBS PG Institute of Biomedical Sciences and Research, Dehradun, Uttarakhand, India; 1 Department of Pharmaceutics, Luqman College of Pharmacy, Gulbarga, Karnataka, India; 2 Department of Pharmaceutical Chemistry, HKE’s College of Pharmacy, Gulbarga, Karnataka, India

**Keywords:** Polyacrylic acid, stimuli-sensitive hydrogels, timolol maleate, viscosity

## Abstract

**Background:**

Stimuli-sensitive hydrogels are three-dimensional, hydrophilic, polymeric networks capable of imbibing large amounts of water or biological fluids on stimulation, such as pH, temperature and ionic change.

**Aim:**

To develop hydrogels that are sensitive to stimuli, i.e. pH, in the cul-de-sac of the eye for providing a prolonged effect and increased bioavailability with reduction in frequency of administration.

**Materials and Methods:**

Hydrogels were formulated by using timolol maleate as the model drug, polyacrylic acid as the gelling agents, hydroxyl ethyl cellulose as the viscolizer and sodium chloride as the isotonic agent. Stirring of ingredients in pH 4 phosphate buffer at high speed was carried out. The dynamic dialysis technique was used for drug release studies. *In vivo* study for reduction in intraocular pressure was carried out by using albino rabbits.

**Statistical Analysis:**

Drug release studies data were used for statistical analysis in first-order plots, Higuchi plots and Peppas exponential plots. Student t-test was performed for *in vivo* study.

**Results:**

Viscosity of the hydrogel increases from 3.84 cps to 9.54 cps due to change in pH 4 to pH 7.4. The slope value of the Peppas equation was found to be 0.3081, 0.3743 and 0.2964. Up to 80% of drug was released in an 8 h drug release study. Sterile hydrogels with no ocular irritation were obtained.

**Conclusions:**

Hydrogels show increase in viscosity due to change in pH. Hydrogels were therapeutically effacious, stable, non-irritant and showed Fickian diffusion. *In vivo* results clearly show a prolonged reduction in intraocular pressure, which was helpful for reduction in the frequency of administration.

Stimuli-sensitive hydrogels are three-dimensional, hydrophilic, polymeric networks capable of imbibing large amounts of water or biological fluids.[1,2] These hydrogels exhibit a thermodynamic capability with water, which allow them to swell in aqueous media.[1–3] Hydrogels show a swelling behavior dependent on the external environment. These polymers are physiologically responsive hydrogels,[4] where polymer complexes can be swollen as a result of the changing external environment. These systems show drastic changes in their swelling behavior. Stimuli-sensitive hydrogels have been stimulated by pH, ionic strength, temperature and electromagnetic radations.[4] Some stimuli-sensitive polymers contain pendant acidic or basic groups that either accept or release protons in response to stimuli, i.e. changes in environmental pH.[5] Swelling of hydrogels increases as the external pH increases in case of weakly acidic (anionic) groups, but decreases if the polymer contains weakly basic (cationic) groups. Most of the anionic pH-sensitive polymers are based on polyacrylic acid (carbopol) or its derivatives.[6]

In the ophthalmic drug-delivery systems, the physiological constraints imposed by the protective mechanism of the eye lead to the low absorption of drugs resulting in the short duration of action. After instillation of drug solution in the eye cavity, the effective tear drainage and blinking action of the eye results in 10-times reduction in the drug concentration within 4-20 min.[7] Due to tear drainage, most of the administered dose passes via the nasolacrimal duct into the gastrointestinal tract (GI) tract, leading to the side-effects. The normal volume of tear in the eye is 7 µl (accommodating capacity) whereas a nonblinking eye can accommodate a maximum of 30 µl biological fluid.[8] The blinking eye can hold only 10 µl, both tears and externally added solution, whereas, usually, the size of a single drop instilled is up to 50 µl. Thus, most of the instilled eye drop is lost leading to limited pre-corneal residence time.

Some of the related work on stimuli-sensitive hydrogels are sol-gel transition on ocular surface by temperature-sensitive polymer (pluronics),[9] pH-triggered systems, including cellulose acetate hydrogen phthalate latex,[10,11] and ion-activated systems, including gelrite,[12] gellan,[13] carbopol/pluronics.[14]

Over the past several years, great attention has been focused on the development of controlled and sustained ophthalmic drug-delivery systems. The goal in designing these systems is to reduce the frequency of dosing or to increase effectiveness of the drug by localization at the site of action and providing uniform drug delivery. In the present research work, stimuli-sensitive hydrogels, i.e. pH-sensitive hydrogels containing timolol maleate (anti-glaucoma agent), polyacrylic acid (gelling agent) and hydroxyl ethyl cellulose (HEC) (thickening agent) were prepared and evaluated.

## Materials and Methods

Timolol maleate was provided by FDC Pvt. Ltd., Mumbai, India. Carbopol 934p was provided by Noveon Polymers, Arihant Trading Co., Mumbai, India. Viscolizers, i.e. HEC, were made available by S.D. Fine – Chem. Ltd., Biosar. Triethanolamine, sodium hydroxide flakes and sodium chloride were provided by S.D. Fine Chem. Pvt. Ltd., Mumbai, India. All the reagents were of analytical grade.

Animals used for the study are albino rabbits. Six rabbits of both sexes weighing between 1.8 kg and 2.2 kg were selected. The procedure involving animals was reviewed and approved by the Animal Ethics Committee Committee for the Purpose of Controlled Supervision of Experimental Animals (CPCSEA).

**Preparation of hydrogels:** Timolol maleate, an anti-glaucoma agent, along with polyacrylic acid as gelling agent, i.e. carbopol 934p, and HEC as thickening agent and sodium chloride as isotonic agent were formulated together to attain the ophthalmic dosage forms. The excipients included in the stimuli-sensitive hydrogel to perform different functions were benzalkonium chloride as a preservative,[15] ethylene diamine tetraacetic acid (EDTA) as chelating agent, sodium chloride as tonicity contributor[16] and HEC as thickening agent.

Weighed quantities of timolol maleate, benzalkonium chloride, EDTA and sodium chloride [Table 1] were dissolved in the pH 4 phosphate buffers under aseptic conditions in three different samples (T_1_, T_2_, T_3_). Polyacrylic acid (carbopol 934p) was slowly added with continuous stirring at a speed of 1,500-2,000 rpm to minimize the formation of the lumps of undispersed mass. HEC was added with slow stirring to avoid foam formation. Stirring was continued until a clear dispersion was formed.

**Table 1:** List of ingredients of stimuli-sensitive hydrogels in three different samples

Ingredient	Concentration (%w/v)		
	T_1_	T_2_	T_3_
Timolol maleate	0.25	0.25	0.25
Benzalkonium chloride	0.01	0.01	0.01
EDTA	0.1	0.1	0.1
Sodium chloride	0.9	0.9	0.9
Carbopol 934p	0.30	0.30	0.30
Hydroxy ethyl cellulose	–	0.4	0.5
pH 4 buffer	150 ml	150 ml	150 ml

**Viscosity studies:** Viscosity determination of the prepared hydrogels was carried out using a Brookfield’s viscometer LVDV II^+^. (Brookfield Engineeering Laboratories Inc, Commerce Boulevard, Middleboro, MA, USA). The correct viscosity of the hydrogels was noted at different spindles (10, 30, 50, 60 and 100). The maximum percent torque value shown at a specific spindle is considered as optimum viscosity [Table 2]. The literature suggests that the viscosity value in the range of 15 cps to 50 cps[17] significantly improves the contact time of the formulation on the corneal surface.

**Table 2:** Viscosity (cps) of the stimuli-sensitive hydrogels at pH 4.0 phosphate buffer and pH 7.4 phosphate buffer in three different samples

	pH	T_1_	T_2_	T_3_
Viscosity (cps)	4.0	3.84	5.40	5.94
	7.4	6.72	9.06	9.54

**Drug-polymer interaction studies:** Drug-polymer interactions were carried out by infrared spectral analysis. Infrared spectra of timolol maleate pure drug and hydrogels were scanned using a Perkins Elmer 1600 FTIR (Perkin Elmer, Inc., Waltham, Massachusetts, USA) by the thin film method.

***In vitro* drug release study:** Drug release from the hydrogels was determined by the diffusion process. One milliliter of the hydrogel was kept in the donor compartment over a cellophane membrane that was rinsed and soaked for 24 h in the diffusion medium. The donor compartment was immersed in the receptor compartment containing 50 ml of the phosphate buffer of pH 7.4. The beaker containing diffusion medium (receptor compartment) was maintained at 37°, with constant stirring at 22 rpm[18] using a magnetic stirrer. One-milliliter aliquots were withdrawn from the diffusion medium every hour for the past 8 h and the same quantity of fresh, pre-warmed diffusion medium was replaced. The samples withdrawn were analyzed spectrophotometrically at 294 nm[19] for timolol maleate using a Shimazdu double-beam UV-Visible spectrophotometer. (Shimadzu corporation, kanda-nithikicho 1- chome, chiyoda- ku, Tokyo, Japan)

**Sterility testing:** Sterility test of the stimuli-sensitive hydrogels was performed for aerobic and anaerobic bacteria and fungi using an alternative thioglycolate medium and soyabean casein digest medium. The positive control (growth promotion) and negative control (sterility) test were also carried out. *Bacillus subtilis, Bacteriodes vulgatus* and *Candida albicans* were used as test organisms in the aerobic bacteria, anaerobic bacteria and fungi test, respectively. Incubation was carried out in all cases and growth was observed.

***In vivo* evaluation:** Induction of glaucoma in the rabbit eye was carried out by the method of Bonomi *et al*.[20] In this method, six albino rabbits of both sexes weighing between 1.8 kg and 2.2 kg were used. The animals were housed in standard cages. They were maintained under controlled room temperature (22 ± 2°C) and humidity (55 ± 5%), with 12:12 h light and dark cycle. All the animals were provided with commercially available diet and water ad *libitum* and comparative evaluation was also performed with commercial available eye drops. Increase in the intraocular pressure was achieved by subconjuctival injection[21] of betamethasone 4 mg/ml every week for 4 weeks [Fig. 1a]. Lowering of intraocular pressure was measured using a Schiotz tonometer (Rudolf Riester GmbH & Co., K.G. Postfach 35, Jungingen, Germany). The stimuli-sensitive hydrogels were evaluated for decrease in intraocular pressure in the rabbit eye model. Three rabbits were used for hydrogel instillation and three were used for commercial eye drop instillation. Commercial eye drop used was Iotim (FDC Ltd. Baddi, Himachal Pradesh, India). Drug instillation was performed once during the initial phase with a specified amount of drug. Intraocular pressure measurement was performed after every half hour till 2 h and then after every 1 h.

**Figure 1a:**
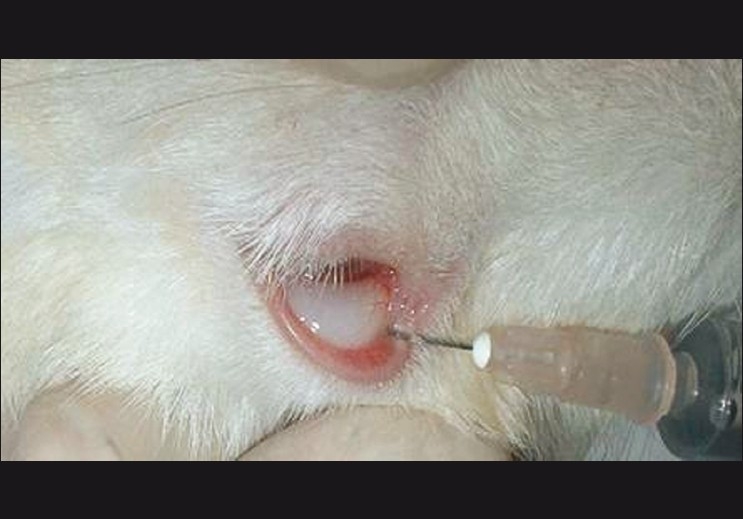
Sub conjuctival injection of betamethasone in Rabbit eye for induction of Glaucoma

Eye irritation studies: A modification of the scoring system of Friendenwald, Hughes and Herrmann (modified Draize technique)[22] was used. In this, injuries to the cornea, conjunctiva, palpebral mucosae and the iris were scored separately.

In this study, six albino rabbits of both sexes weighing 1.8-2.2 kg were used for the study. 0.1 ml of the stimuli-sensitive hydrogel was instilled in the conjunctival sac of each rabbit and readings were made at 1, 24 and 48 h after instillation of the hydrogel into the eye, and were evaluated on the guidelines of scale of weighted scores for grading the severity of ocular lesions. In all three sections for the 1^st^, 24^th^ and 48^th^-h observations, the scores given to the rabbits were less than the maximum total scores [Table 3]. The cornea, iris and conjunctivae were evaluated for several parameters such as opacity and its degree of density, opaqueness (in case of cornea), swelling (in case of iris), redness, chemosis, discharge (in case of conjunctivae) and allotted with maximum scores of 80, 10 and 20, respectively. The total maximum score was 110.

**Table 3:** Eye irritation studies

Group	Tissues	Total scores	Total maximum scores
Section I	Cornea	05	80
Section II	Iris	05	10
Section III	Conjunctivae	06	20

## Results

Rheological studies: Viscosity of the hydrogel at pH 4 phosphate buffer was found to lie between 3.84 cps and 5.94 cps, i.e. less viscous, and at pH 7.4 the phosphate buffer viscosity increased up to 6.72-9.54 cps.

Drug-polymer interaction studies: Timolol maleate in its infrared spectrum exhibits a strong peak at 3445 cm^-1^, indicating the presence of - OH group. Presence of the – NH group was supported by exhibition of a peak to the main peak around 2,100 cm^-1^. More than one C=N bond absorption peak was due to the thiadizole moiety of the heterocyclic ring system of the drug molecule. When pure drug was formulated with carbopol 934p and viscolizers, the spectrum obtained by this hydrogel exhibited a broad absorption peak from 3,050 cm^-1^ to 3,500 cm^-1^, indicating the participation of the alkali hydroxyl in forming gel preparation. The increased viscosity leads to a broadening of the peak. The spectral data suggest that the intactness of the thiadizole ring structure of timolol maleate, indicated by the absence of additional peaks that confirm the opening of the thiadizole ring, was not taking place. Hence, the drug was not reacting with the polymers used in the stimuli-sensitive hydrogels.

*In vitro* drug release study: The drug release data were plotted for cumulative percent drug released vs. time, log cumulative percent drug released-retained vs. time (first-order plot), cumulative percent drug released vs. square root of time (Higuchi plot), log cumulative percent drug release vs. log time (Peppas equation) [Figs. 1–4] To know precisely the rate of drug release, the basic *in vitro* data was plotted according to the first-order kinetics. The results show that the plots are fairly linear and that the degree of linearity was ascertained by carrying out regression analysis and regression coefficient values. To ascertain the drug release mechanism, the hydrogels were plotted for Higuchi diffusion plots. The plots were fairly linear and the drug release mechanism was found to be diffusion controlled. In case of the Peppas exponential equation, the slope values of the Peppas equation were found to be 0.3081, 0.3743 and 0.2964, i.e. <0.5.

**Figure 1:**
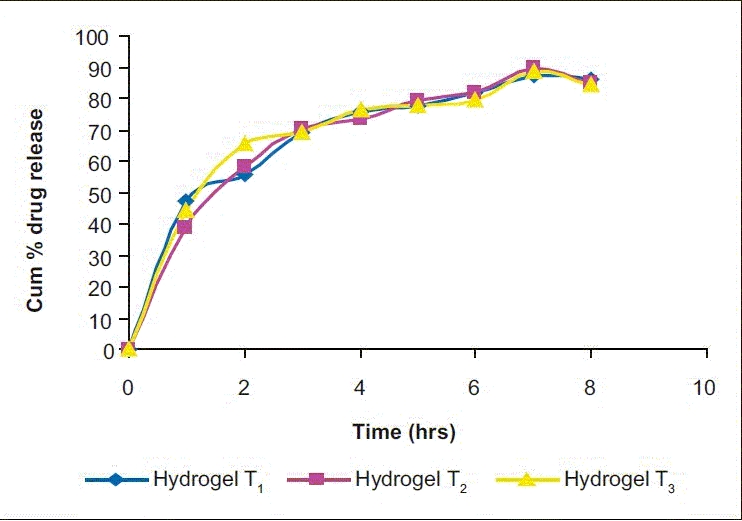
Cumulative % drug release vs time in three different samples

**Figure 2:**
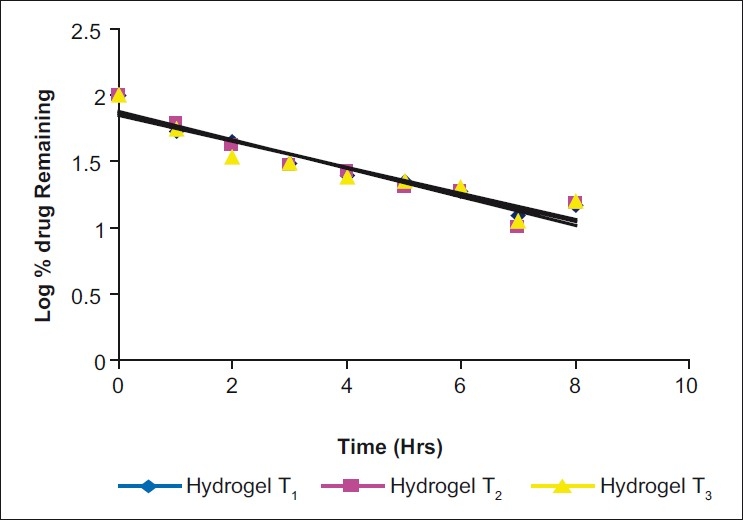
First order plots (Log cumulative % drug released-retained vs time) in three different samples

**Figure 3:**
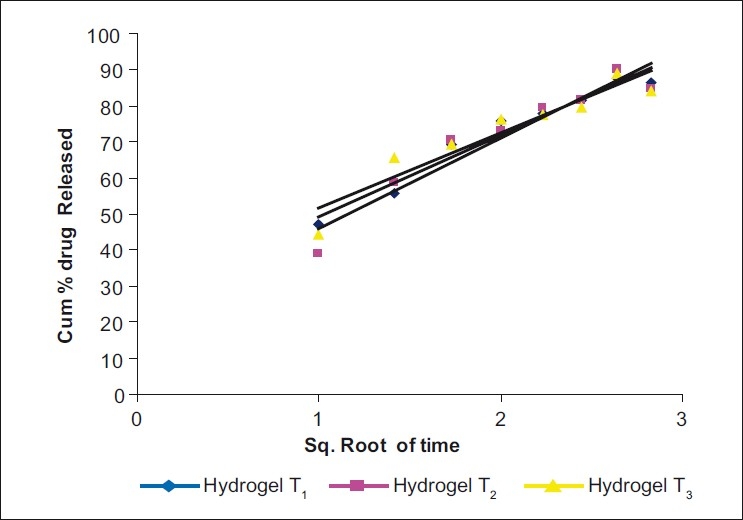
Higuchi diffusion plots (Cumulative % drug released vs square root of time) in three different samples

**Figure 4:**
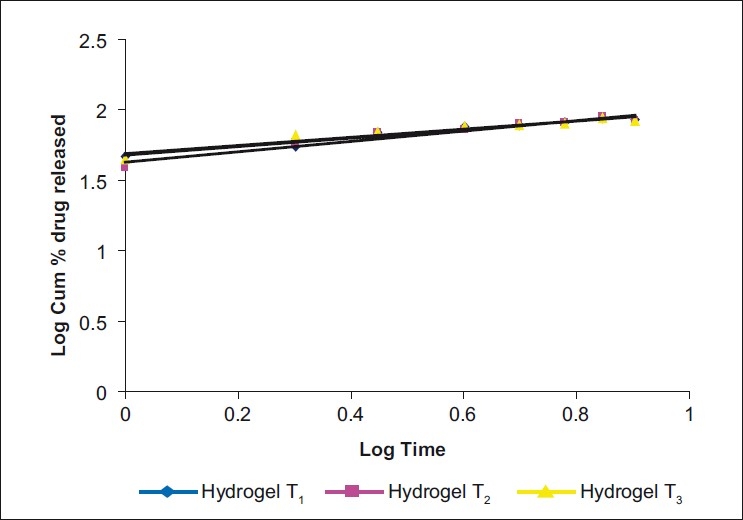
Peppa’s exponential plots (Log cumulative % drug released vs log time) in three different samples

Sterility testing : The sterility test showed that the stimuli-sensitive hydrogels pass the sterility test as there was no evidence of growth in the negative control test tubes.

**Figure 5:**
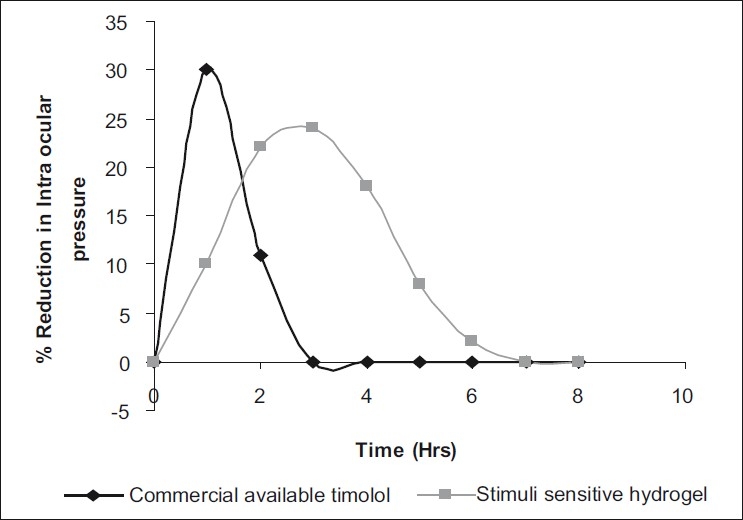
*In vivo* study of intraocular pressure over time

*In vivo* evaluation: The marketed eye drops suddenly lowered the intraocular pressure to a minimum and, afterwards, there was a sudden increase in the intraocular pressure [Fig. 5] to the original reading, whereas the stimuli-sensitive hydrogels lowered the intraocular pressure slowly to the original and, thereafter, a gradual increase in the intraocular pressure was observed. Thus, a sustained effect was maintained with the stimuli-sensitive hydrogels.

Ocular safety/eye irritation studies: In the case of the cornea, the total score was found to be 5, with score 1 in opacity and score 1 in area of cornea involved. In case of the iris, the score was found to be 5, with score 1 due to swelling. In case of the conjunctivae, the score was found to be 6, with individual scores of 1 due to the parameter of redness, chemosis and discharge. The total score was found to be 5 + 5 + 6 = 11. Score 11 was obtained from the maximum score.

## Discussion

Stimuli-sensitive hydrogels were liquid at pH 4 phosphate buffers and underwent rapid swelling or thickening at pH 7.4 phosphate buffer. The hydrogels provide a sustained drug release of up to 90% over an 8-h period. Viscosity of the prepared hydrogels lies in the optimum range, i.e. 25 cps, at the pH 4 phosphate buffer and up to 50 cps at the pH 7.4 phosphate buffers. Infrared spectroscopy shows that there was no interaction of the thiadizole ring, which indicates that the drug and the polymer were not reacting together. All the stimuli-sensitive hydrogels passed the test for sterility and growth was not observed.

Drug-release studies data show a fairly linear curve in the first-order plots, Higuchi plots and Peppas exponential plots. The slope values of the Peppas equation were found to be 0.3081, 0.3743 and 0.2964, hence following Fickian diffusion. Stimuli-sensitive hydrogels were safe and therapeutically efficacious and provided increased bioavailability and prolonged therapeutic response. Ocular irritation results show that irritation was not observed due to the sensitive ocular tissues by the stimuli-sensitive hydrogels, and was safe in nature. In a comprehensive review of the Draize test, it was noted that the anatomy and biochemistry of the rabbit eye are not the same as that of the human eye, and that there were numerous physiological reasons, including low tear production, blink frequency and ocular surface area, and that such a test might not predict human effect.[23] Yark and Steiling[24] stressed the need to validate the Draize test against controlled human eye data, but noted that “there is no adequate data.” *In vivo* results clearly show that the hydrogels provide a better therapeutic effect in the lowering of intraocular pressure for a prolonged period of time in comparison to the marketed conventional dosage form.
